# Association between Exercise-Induced Changes in Cardiorespiratory Fitness and Adiposity among Overweight and Obese Youth: A Meta-Analysis and Meta-Regression Analysis

**DOI:** 10.3390/children7090147

**Published:** 2020-09-21

**Authors:** Antonio García-Hermoso, Mikel Izquierdo, Alicia M. Alonso-Martínez, Avery Faigenbaum, Jordi Olloquequi, Robinson Ramírez-Vélez

**Affiliations:** 1 Navarrabiomed, Complejo Hospitalario de Navarra (CHN), Universidad Pública de Navarra (UPNA), IdiSNA, 31008 Pamplona, Navarra, Spain; mikel.izquierdo@gmail.com (M.I.); robin640@hotmail.com (R.R.-V.); 2 Facultad de Ciencias Médicas, Laboratorio de Ciencias de la Actividad Física, el Deporte y la Salud, Universidad de Santiago de Chile, USACH, Santiago 71783-5, Chile; 3 Department of Health Sciences, Public University of Navarra, 31006 Pamplona, Navarra, Spain; aliciamaria.alonso@unavarra.es; 4 CIBER of Frailty and Healthy Aging (CIBERFES), Instituto de Salud Carlos III, 28001 Madrid, Spain; 5 Department of Health and Exercise Science, The College of New Jersey, Ewing, NJ 08628, USA; faigenba@tcnj.edu; 6 Instituto de Ciencias Biomédicas, Facultad de Ciencias de la Salud, Universidad Autónoma de Chile, Talca 3460000, Chile; jolloquequig@uautonoma.cl

**Keywords:** cardiorespiratory fitness, fatness, physical activity, obesity

## Abstract

The aim of this study was to determine the minimum change in cardiorespiratory fitness (CRF) required to reduce adiposity (percent body fat) in exercise programs for overweight and obese youth. Studies were identified through a systematic search of five databases. Studies were limited to randomized controlled trials (RCTs) of exercise training (e.g., aerobic, strength, concurrent) that assessed percent body fat and CRF for both exercise and control groups in overweight and obese children and adolescents. A series of meta-regressions were conducted to explore links between change in CRF (maximum oxygen consumption, ml/kg/min) and change in percent body fat. Twenty-three RCTs were included (*n* = 1790, 59% females). Meta-regression analysis suggested that increases of at least 0.38 mL/kg/min in CRF (*p* < 0.001) were considered to be a clinically important reduction of percent body fat (−2.30%, 95% confidence interval −3.02 to −1.58; *p* < 0.001; *I*^2^ = 92.2%). Subgroup analysis showed that increases of at least 0.17 mL/kg/min in CRF favored a reduction of percent body fat of −1.62% (95% confidence interval −2.04 to −1.20; *p* < 0.001; *I*^2^ = 69.9%). In conclusion, this change in CRF could be considered by pediatric researchers, youth fitness specialists, and health care providers to determine the effectiveness in body fat reductions through exercise.

## 1. Introduction

Childhood obesity is one of the most prevalent chronic health conditions and has become an epidemic in modern-day society [1]. Obesity in children and adolescents has deleterious consequences, both in the short and long-term. More immediate health risks of childhood obesity are associated with an increased risk for prediabetes, musculoskeletal problems, and pulmonary (e.g., obstructive sleep apnea, and asthma) and psychosocial (e.g., poor self-esteem, anxiety, depression, and social dysfunction) abnormalities [2]. Over the long-term, adverse health outcomes include a higher risk for cardiovascular (e.g., hypertension and dyslipidemia) and endocrine (e.g., type 2 diabetes and fatty liver disease) conditions, and an increased risk of obesity in adulthood [3]. Overall, this context highlights the need to develop effective management interventions to slow or stop the progression of this chronic disease and to reduce adiposity among the young population.

One of the principal strategies for the treatment of pediatric obesity is adequate physical activity [2]. Regular participation in exercise training can exert a positive effect on health and is an important component of youth weight management programs [2]. A recent network meta-analysis by Kelley et al. [4] concluded that regular exercise, mainly aerobic and concurrent (aerobic and strength exercise) training, can lead to clinical improvements in adiposity outcomes in youth with overweight and obesity, confirming a previous meta-analysis [5]. 

Cardiorespiratory fitness (CRF) is considered an important health indicator among youth [6]. Specifically, a higher level during childhood and adolescence is associated with lower body mass index (BMI) and reduced body fat in later life, in addition to other health benefits [6]. A previous meta-analysis has shown that programs based on aerobic exercise have a moderate positive effect on CRF among obese children [7]. However, the level of improvement in CRF parameters needed to produce clinically significant reductions in obesity in youth is currently unknown. It is important to underpin that when assessing exercise interventions designed to manage overweight and obesity in children and adolescents, it is essential to recognize the real purpose of measures such as CRF surrogates (i.e., reduction of body fat). Indeed, to the best of our knowledge, there has been no systematic quantification of the required increment in CRF levels to reduce adiposity in pediatric populations with overweight/obesity through exercise interventions. Thus, the aim of the present study was to determine, through meta-regression, the minimum change in CRF required to reduce percent body fat in exercise programs in overweight and obese children and adolescents.

## 2. Materials and Methods

The study was conducted and reported according to PRISMA Statement Checklist guidelines [8]. Protocol was not previously registered. The entire process from literature selection to data extraction was performed independently by two authors. Any disagreements were resolved through consultation with a third researcher.

### 2.1. Eligibility Criteria

To be eligible for inclusion in the systematic review, randomized controlled trials (RCTs) needed to meet the following criteria established a priori: (i) children and adolescents classified as overweight or obese (as defined by the authors) aged 3 to 18 years (mean age); (ii) RCTs which include a comparison group (i.e., control group) without structured type of exercise or calorie restriction; (iii) exercise interventions (e.g., aerobic, strength, and concurrent) had to report data of percent body fat and CRF estimated as maximum oxygen consumption (VO2max or peak) in mL/kg/min. 

### 2.2. Information Sources and Search

Ovid, MEDLINE, EMBASE, Cochrane Controlled Trials Registry, and SPORTDiscus electronic databases were searched for studies from inception to 30 November 2019. The MeSH terms used were: (“Obesity” and “Overweight” OR), (“Adipose Tissue”, “Anthropometric”, “Body Composition”, “Body Fat Distribution”, and “Body Mass Index” OR) and combined with aerobic *, resistance *, concurrent *. Additionally, searches were limited to youth between 3 and 18 years old and randomized controlled trials (RCT) (i.e., article types). Cross referencing was also used by examining the reference lists of articles that met the inclusion criteria.

### 2.3. Study Selection and Data Collection Process

The first and second authors independently extracted the following data from the identified studies: characteristics of the studies (i.e., authors name, sample size, country, duration of intervention, and publication year); characteristics of participants, age and sex; characteristics of the exercise intervention (i.e., frequency, duration, adherence and type (aerobic, strength, or concurrent)); and analysis and results (percent body fat and CRF). 

### 2.4. Risk of Bias in Individual Studies

The quality of the studies was evaluated using the Physiotherapy Evidence Database (PEDro) criteria (www.pedro.org.au) [9]. This tool is a 11-item scale designed for rating the methodological quality of RCTs. 

### 2.5. Synthesis of Results

All analyses were conducted using the random-effects inverse-variance model with the Hartung–Knapp–Sidik–Jonkman variance estimator based on DerSimonian–Laird estimate of tau and carried out using the STATA software (version 13.1; StataCorp, College Station, TX, USA). Changes in percent body fat and CRF for RCTs were calculated by subtracting change differences between the exercise and control groups, using the pooled standard deviation (SD) of change in both groups. If change scores SD were not available, they were calculated from 95% confidence intervals (CI) for either change outcome or exercise training effect differences as well as pre-SD and post-SD values [10]. Second, meta-regression analysis using method of moments was conducted to quantify the relationships between mean change in percent body fat and mean change in CRF. The smallest reduction in CRF associated with a reduction in percent body fat was determined as the smallest reduction in CRF with an associated 95% prediction interval wholly below zero. Heterogeneity across RCTs was calculated using the inconsistency index (*I*^2^) [11]. Finally, a subgroup analysis was conducted using mean change in percent body fat assessed by dual-energy x-ray absorptiometry (DXA) or magnetic resonance imaging (MRI) and CRF assessed by direct gas analysis.

## 3. Results

### 3.1. Study Selection

**Figure 1:**
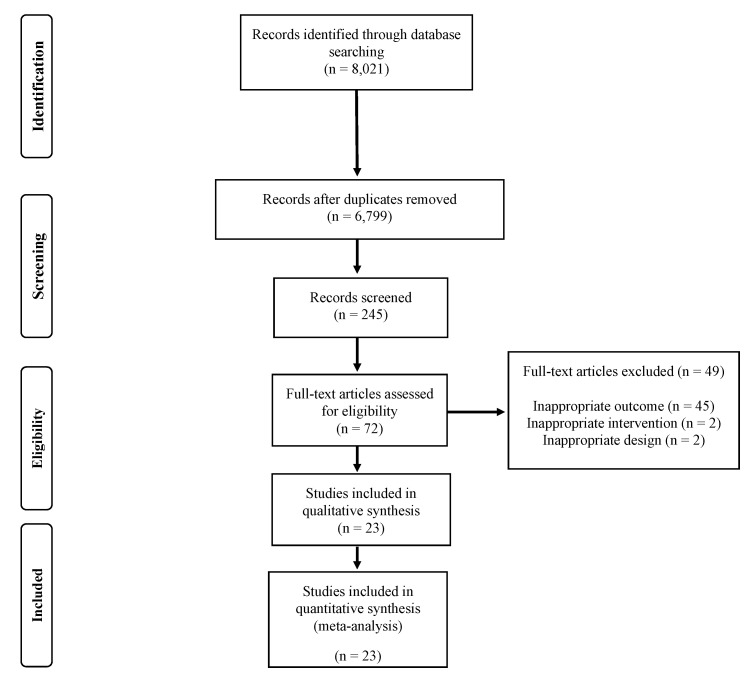
PRISMA flow diagram.

A total of 23 RCTs were included in meta-regression analysis [12,13,14,15,16,17,18,19,20,21,22,23,24,25,26,27,28,29,30,31,32,33,34]. The PRISMA flow diagram is shown in Figure 1.

### 3.2. Study Characteristics

A summary of the 23 studies included in this study is listed in Table 1. RCTs included in the meta-regression involved a total of 1790 children and adolescents (59% females), ranging from 18 [35] to 322 [17] participants per study. All studies included males and females with the exception of seven studies that included only females [15,20,21,22,27,29,35] or three only males [23,25,35]. 

Studies included aerobic (*n* = 18 intervention groups), strength (*n* = 5), and concurrent (*n* = 5) interventions with an average duration of 12 weeks, ranging from eight [14] to 36 [30] weeks. Only nine RCTs included the adherence to the training programs (>50%) [12,15,16,18,19,28,32,33,34].

Studies assessed the percent body fat by DXA [14,18,23,25,26,27,28,30,32,33,34], MRI [12,15,16,19], bioelectrical impedance analysis [17,20,21,29,31,35] or skinfolds thickness [22]. Regarding CRF, most RCTs used maximal or submaximal tests on a cycle ergometer or treadmill with spirometry to determine oxygen consumption. 

### 3.3. Synthesis of Results

**Figure 2:**
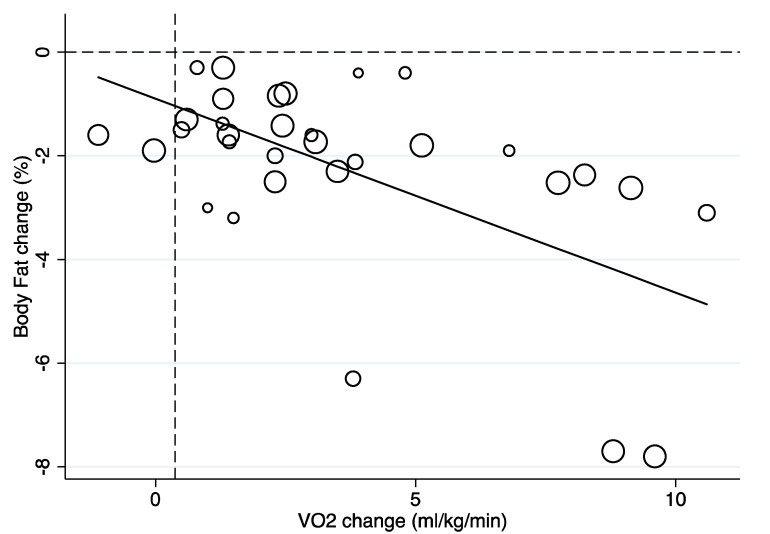
Meta-regression line showing the relationship between change in percent body fat and CRF across the randomized controlled trials (RCTs) included. The circles represent the study results (i.e., the mean difference in percent body fat) analyzed for each study, with the size of the circles representing the precision of the effect size change in percent body fat.

Meta-regression analysis suggested that increases of at least 0.38 mL/kg/min in CRF (*p* < 0.001) (Figure 2) favored a reduction of percent body fat (−2.30%, 95% CI −3.02 to −1.58; *p* < 0.001; *I^2^* = 92.2%; Figure S1). Variance explained by the change in CRF was 32.5% (i.e., R-square). However, the heterogeneity was high (*I^2^* = 88.5%). 

**Figure 3:**
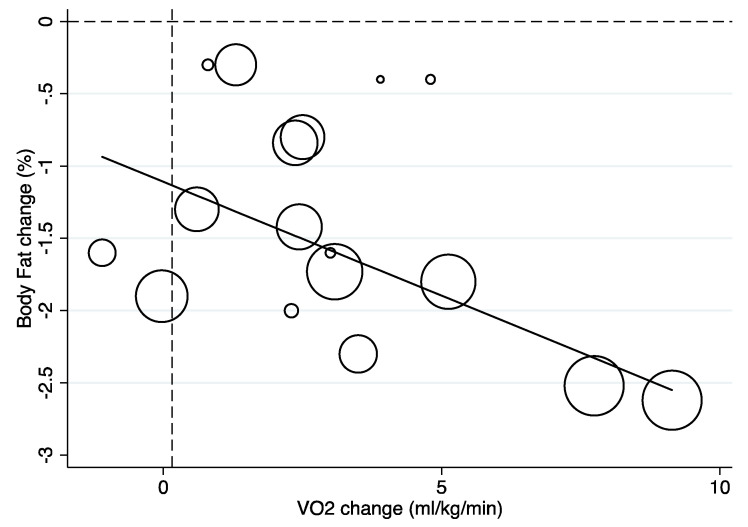
Meta-regression line showing the relationship between change in percent body fat assessed by dual-energy X-ray absorptiometry or magnetic resonance imaging and CRF assessed by direct gas analysis across the RCTs included. The circles represent the study results (i.e., the mean difference in percent body fat) analyzed for each study with the size of the circles representing the precision of the effect size change in percent body fat.

Subgroup analysis showed that increases of at least 0.16 mL/kg/min (*I^2^* = 33.9%) in CRF assessed by direct gas analysis (Figure 3) favored a reduction of percent body fat (by DXA or MRI) of −1.62% (95% CI −2.04 to −1.20; *p* < 0.001; *I^2^* = 69.9 Figure S2). Variance explained by the change in CRF was 75.8%.

### 3.4. Risk of Bias within Studies

All included RCTs had random allocation between groups and provided points and estimates of variability. Six RCTs performed concealed allocation [16,17,26,32,33,34]. Blinding of youth and therapists was not possible because of the nature of exercise interventions. Assessor blinding was implemented in three of the included trials [12,14,34]. Complete details are reported in Table S1.

## 4. Discussion

The objective of this study was to establish the minimum change in CRF (estimated as maximum oxygen consumption (VO2max or peak) in ml/kg/min) needed to reduce adiposity in exercise programs for overweight/obese children and adolescents. The meta-regression analysis indicated that increases of at least 0.38 mL/kg/min in CRF favored a clinically important reduction of at least 2.30% in percent body fat [36]. Thus, our study suggests that exercise interventions should aim to increase CRF by at least this amount to be confident of achieving a reduction of percent body fat in overweight and obese youth.

It is well established that body fat is a strong risk factor for obesity-related comorbidities in youth [36]. Several studies have reported the beneficial effects of exercise alone for clinically relevant improvements in body composition and metabolic parameters in youth with obesity [4,5], because fat is a key factor in metabolic complications [36]. The present meta-regression analysis also indicates a negative relationship between CRF and change in percent body fat. Specifically, CRF explains 32.5% of variance with results showing an increase of 1 metabolic equivalent of task (MET) in VO2 of the children and adolescents (mean difference = 3.52 mL/kg/min, 95% CI 1.94 to 5.11, *p* < 0.001, *I^2^* = 97.7; Figure S3) through exercise programs, which confirms a previous meta-analysis in youth population with obesity [7]. 

The possible mechanisms that could explain our findings have been investigated in-depth [37]. Adipose tissue releases interleukins and pro-inflammatory cytokines that influence endocrine physiology by accelerating inflammation and endothelial dysfunction, and reducing insulin clearance in the liver (“the portal theory”) [38]. In addition, it could be hypothesized that higher CRF increases muscular enzymatic function, which improves insulin sensitivity. This improvement could be secondary to the activation of the enzyme 5′-AMP-activated protein kinase (AMPK) and to the reduction of the burden of intracellular lipid accumulation via increased mitochondrial fatty acid oxidation in different tissues [39]. Both physical activity and physical fitness are considered the first line of treatment to reduce adiposity and improve metabolic function, in part by augmenting overall mitochondrial density and oxidative phosphorylation capacity in skeletal muscle by as much as two-fold [40]. This suggests that higher levels of CRF attenuate the increased risk of metabolic disturbance associated with increased adiposity. 

### Strength and Limitations

The main strength of the present study is that it, to the best of our knowledge, is the first to determine the minimum change of CRF necessary to reduce adiposity among overweight and obese youth. However, there are potential limitations. An important limitation is the heterogeneity in CRF protocol tests and body fat assessment. Most of the RCTs used spirometry to determine oxygen consumption; though many others applied field-tests such as the 20-m Shuttle Run [17,22,24]. However, meta-regression analysis using more robust assessments showed higher variance explained and lower heterogeneity between results. A second limitation is that since this is an aggregate data meta-analysis, the potential for ecological fallacy exists. Finally, since the covariate used in our meta-regression was not randomly assigned in the original studies, our meta-regression findings should be considered exploratory. Thus, our findings would need to be tested in original RCTs. 

## 5. Conclusions

The present meta-regression analysis suggests that there is a negative relationship between change in percent body fat and CRF. Specifically, exercise interventions demonstrating an increase of at least 0.38 mL/kg/min in CRF could obtain a clinically important reduction of at least of 2.30% in percent body fat in overweight and obese children and adolescents, a key goal of weight management interventions through exercise. Therefore, this change in CRF could be considered by pediatric researchers, youth fitness specialists, and health care providers to determine the effectiveness in body fat reductions through exercise.

## Figures and Tables

**Table 1:** Summary of included studies.

Author, Year, Country	Sample, Age (Range or Mean)	Type	Intervention Length (Weeks)	Frequency (Sessions/Week)	Duration (min)	Adherence (%)	Percent Body Fat Assessment	Cardiorespiratory Fitness Assessment
Alberga 2016 [12]/Sigal 2014 [13], Canada	304 (70% girls), 14–18 years old	AE, ST, CT	22	4	20–45	56–64	Magnetic resonance imaging	Treadmill test. Oxygen consumption was measured by indirect calorimetry
Alves et al. 2019 [24], Portugal	40 (NR), 12–15 years old	CT	10	2–3	60	NR	Standard formula	20-m Shuttle Run Test
Berntsen 2010 [28], Norway	60 (51% girls), 7–17 years old	CT	20	2	60	60	Dual-energy X-ray absorptiometry	Treadmill test. Oxygen consumption was measured by indirect calorimetry
Bharath 2018 [29], USA	40 (100% girls), 14.7 years old	CT	12	5	60	NR	Bioelectrical impedance meter	Treadmill running test. Maximal heart rate was obtained.
Carrel 2005 [30], USA	50 (48% girls), 12.5 years old	AE	36	5	45	NR	Dual-energy X-ray absorptiometry	4-min submaximal treadmill walk test. Oxygen consumption was measured by indirect calorimetry
Chae 2010 [31], South Korea	38 (45% girls), 9–15 years old	AE	12	2	90	NR	Bioelectrical impedance meter	Treadmill running test. Oxygen consumption was measured by indirect calorimetry
Davis 2012 [32], USA	222 (58% girls), 7–11 years old	AE	13	5	20–40	84	Dual-energy X-ray absorptiometry	Treadmill test. Oxygen consumption was measured by indirect calorimetry
Dias 2018 [33], Australia	99 (53% girls); 7–16 years old	AE	12	3	40–44	56–68	Dual-energy X-ray absorptiometry	Treadmill test. Oxygen consumption was measured by indirect calorimetry
Farpour-Lambert 2009 [34], Switzerland	44 (64% girls), 6–11 years old	CT	12	3	60	83	Dual-energy X-ray absorptiometry	Treadmill test. Oxygen consumption was measured by indirect calorimetry
Kelly 2004 [14], USA	25 (52% girls), 10.9 years old	AE	8	4	30–50	NR	Dual-energy X-ray absorptiometry	Cycle ergometer test. Oxygen consumption was measured by indirect calorimetry
Lee 2013 [15], USA	54 (100% girls), 12–18 years old	AE, ST	12	3	60	95	Magnetic resonance imaging	Treadmill test. Oxygen consumption was measured by indirect calorimetry
Lee 2010 [35], South Korea	18 (100% girls), 16.7 years old	AE	12	4	40–50	NR	Bioelectrical impedance meter	Åstrand protocol with a cycle ergometer
Lee 2012 [16], USA	45 (0% girls), 12–18 years old	AE, ST	12	3	60	99	Magnetic resonance imaging	Treadmill test. Oxygen consumption was measured by indirect calorimetry
Maddison 2011 [17], New Zeland	322 (27% girls), 10–14 years old	AE	-	-	-	-	Bioelectrical impedance meter	20-m Shuttle Run Test
Mitchell 2002 [19], USA	81 (68% girls), 13–16 years old	AE	32	5	29–43	51–56	Magnetic resonance imaging	Treadmill test. Oxygen consumption was measured by indirect calorimetry
Racil 2013 [20], Tunisia	34 (100% girls), 15.9 years old	AE	12	3	-	NR	Bioelectrical impedance meter	Run test. Oxygen consumption was measured by indirect calorimetry
Racil 2016 [21], Tunisia	68 (100% girls), 16.6 years old	AE, CT	12	3	35	NR	Bioelectrical impedance meter	Run test. Oxygen consumption was measured by indirect calorimetry
Saygın 2011 [22], Turkey	40 (100% girls), 10–12 years old	AE	12	3	50–90	NR	Skinfolds	20-m Shuttle Run Test
Shaibi 2006 [23], USA	22 (0% girls), 15.3 years old	ST	16	2	-	NR	Dual-energy X-ray absorptiometry	Cycle ergometer test. Oxygen consumption was measured by indirect calorimetry
Song 2012 [25], USA	22 (0% girls), 12–13 years old	AE	12	3	50	NR	Dual-energy X-ray absorptiometry	Treadmill test. Oxygen consumption was measured by indirect calorimetry
Sun 2011 [18], China	93 (52% girls), 13.6 years old	AE	10	4	60	57.5	Dual-energy X-ray absorptiometry	Physical Working Capacity at a heart rate of 170 bpm (PWC170)
Vasconcellos 2016 [26], Brazil	30 (20% girls), 12–17 years old	AE	12	3	60	NR	Dual-energy X-ray absorptiometry	Treadmill test. Oxygen consumption was measured by indirect calorimetry
Youssef 2015 [27], France	39 (100% girls), 14–18 years old	AE	12	3	40	NR	Dual-energy X-ray absorptiometry	Treadmill test. Oxygen consumption was measured by indirect calorimetry

AE, aerobic exercise; CT, concurrent training; NR, not reported; PWC170, Physical Working Capacity at a heart rate of 170 bpm; ST, strength training.

## Supplementary Materials

The following are available online at https://www.mdpi.com/2227-9067/7/9/147/s1. Figure S1: The effects of the exercise programs on percent body fat. Squares represent individual studies, and horizontal lines represent 95% confidence limits for individual studies. The diamond represents pooled mean differences. HIIT, high-intensity interval training; MIIT, moderate-intensity continuous training; ST, strength training. Figure S2, The effects of the exercise programs on percent body fat assessed by dual-energy x-ray absorptiometry or magnetic resonance imaging. Squares represent individual studies, and horizontal lines represent 95% confidence limits for individual studies. The diamond represents pooled mean differences. HIIT, high-intensity interval training; MIIT, moderate-intensity continuous training. Figure S3, The effects of the exercise programs on CRF. Squares represent individual studies, and horizontal lines represent 95% confidence limits for individual studies. The diamond represents pooled mean differences. Table S1, PEDro scale assessment.
