# Supplementary material for: Association between Exercise-Induced Changes in Cardiorespiratory Fitness and Adiposity among Overweight and Obese Youth: A Meta-Analysis and Meta-Regression Analysis
Source: Children (Basel). 2020 Sep 21;7(9):147. doi: 10.3390/children7090147 (PMC7552631; doi:10.3390/children7090147)
Supplement: Supplementary file 1 [file children-07-00147-s001.pdf]

Figure S1.

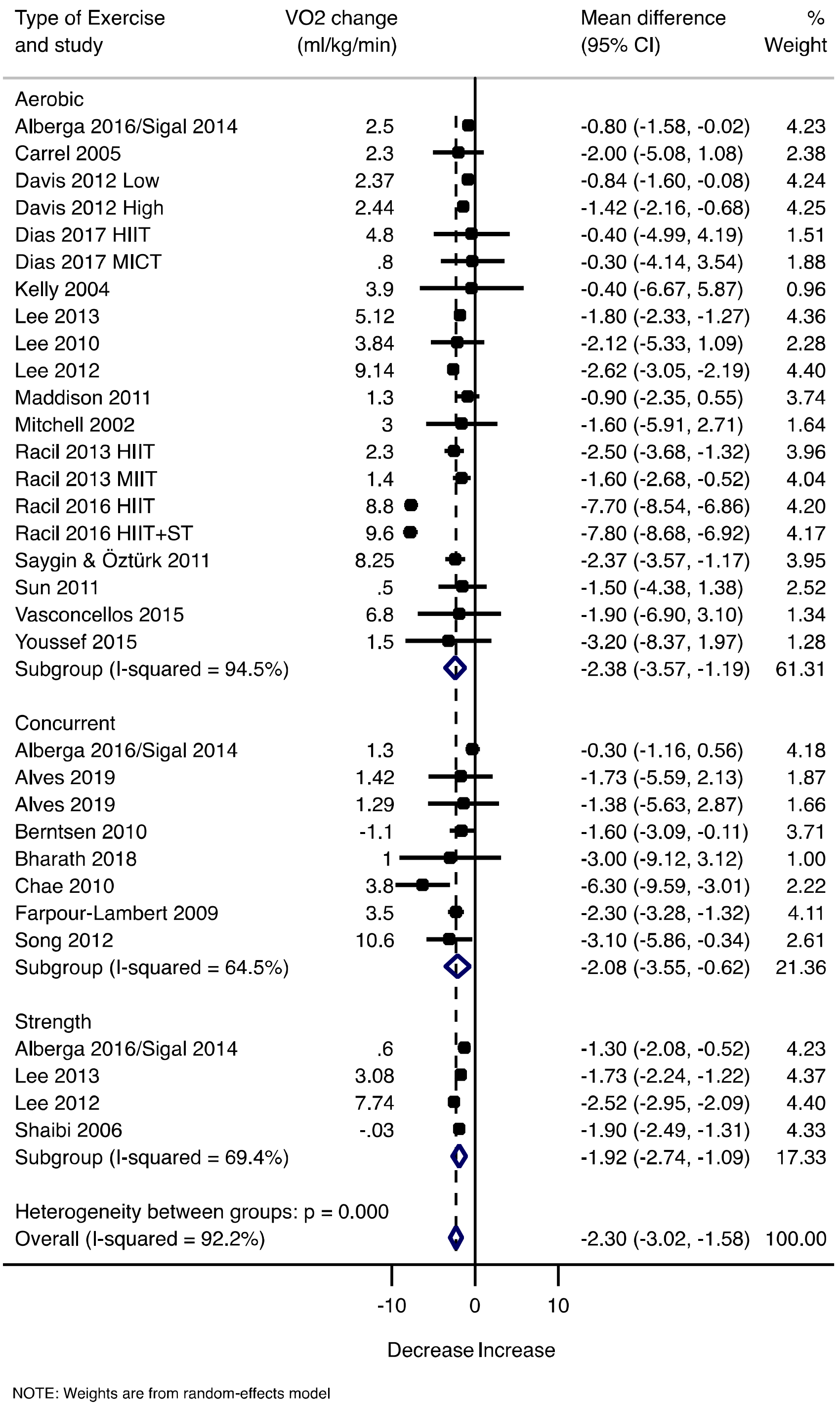
Type of Exercise and study
VO2 change (ml/kg/min)
Mean difference (95% CI)
% Weight
Aerobic
Alberga 2016/Sigal 2014 2.5 -0.80 (-1.58, -0.02) 4.23
Carrel 2005 2.3 -2.00 (-5.08, 1.08) 2.38
Davis 2012 Low 2.37 -0.84 (-1.60, -0.08) 4.24
Davis 2012 High 2.44 -1.42 (-2.16, -0.68) 4.25
Dias 2017 HIIT 4.8 -0.40 (-4.99, 4.19) 1.51
Dias 2017 MICT .8 -0.30 (-4.14, 3.54) 1.88
Kelly 2004 3.9 -0.40 (-6.67, 5.87) 0.96
Lee 2013 5.12 -1.80 (-2.33, -1.27) 4.36
Lee 2010 3.84 -2.12 (-5.33, 1.09) 2.28
Lee 2012 9.14 -2.62 (-3.05, -2.19) 4.40
Maddison 2011 1.3 -0.90 (-2.35, 0.55) 3.74
Mitchell 2002 3 -1.60 (-5.91, 2.71) 1.64
Racil 2013 HIIT 2.3 -2.50 (-3.68, -1.32) 3.96
Racil 2013 MIIT 1.4 -1.60 (-2.68, -0.52) 4.04
Racil 2016 HIIT 8.8 -7.70 (-8.54, -6.86) 4.20
Racil 2016 HIIT+ST 9.6 -7.80 (-8.68, -6.92) 4.17
Saygin & Öztürk 2011 8.25 -2.37 (-3.57, -1.17) 3.95
Sun 2011 .5 -1.50 (-4.38, 1.38) 2.52
Vasconcellos 2015 6.8 -1.90 (-6.90, 3.10) 1.34
Youssef 2015 1.5 -3.20 (-8.37, 1.97) 1.28
Subgroup (I-squared = 94.5%) -2.38 (-3.57, -1.19) 61.31
Concurrent
Alberga 2016/Sigal 2014 1.3 -0.30 (-1.16, 0.56) 4.18
Alves 2019 1.42 -1.73 (-5.59, 2.13) 1.87
Alves 2019 1.29 -1.38 (-5.63, 2.87) 1.66
Berntsen 2010 -1.1 -1.60 (-3.09, -0.11) 3.71
Bharath 2018 1 -3.00 (-9.12, 3.12) 1.00
Chae 2010 3.8 -6.30 (-9.59, -3.01) 2.22
Farpour-Lambert 2009 3.5 -2.30 (-3.28, -1.32) 4.11
Song 2012 10.6 -3.10 (-5.86, -0.34) 2.61
Subgroup (I-squared = 64.5%) -2.08 (-3.55, -0.62) 21.36
Strength
Alberga 2016/Sigal 2014 .6 -1.30 (-2.08, -0.52) 4.23
Lee 2013 3.08 -1.73 (-2.24, -1.22) 4.37
Lee 2012 7.74 -2.52 (-2.95, -2.09) 4.40
Shaibi 2006 -.03 -1.90 (-2.49, -1.31) 4.33
Subgroup (I-squared = 69.4%) -1.92 (-2.74, -1.09) 17.33
Heterogeneity between groups: p = 0.000
Overall (I-squared = 92.2%) -2.30 (-3.02, -1.58) 100.00
-10
0
10
Decrease Increase
NOTE: Weights are from random-effects model

Figure S2.

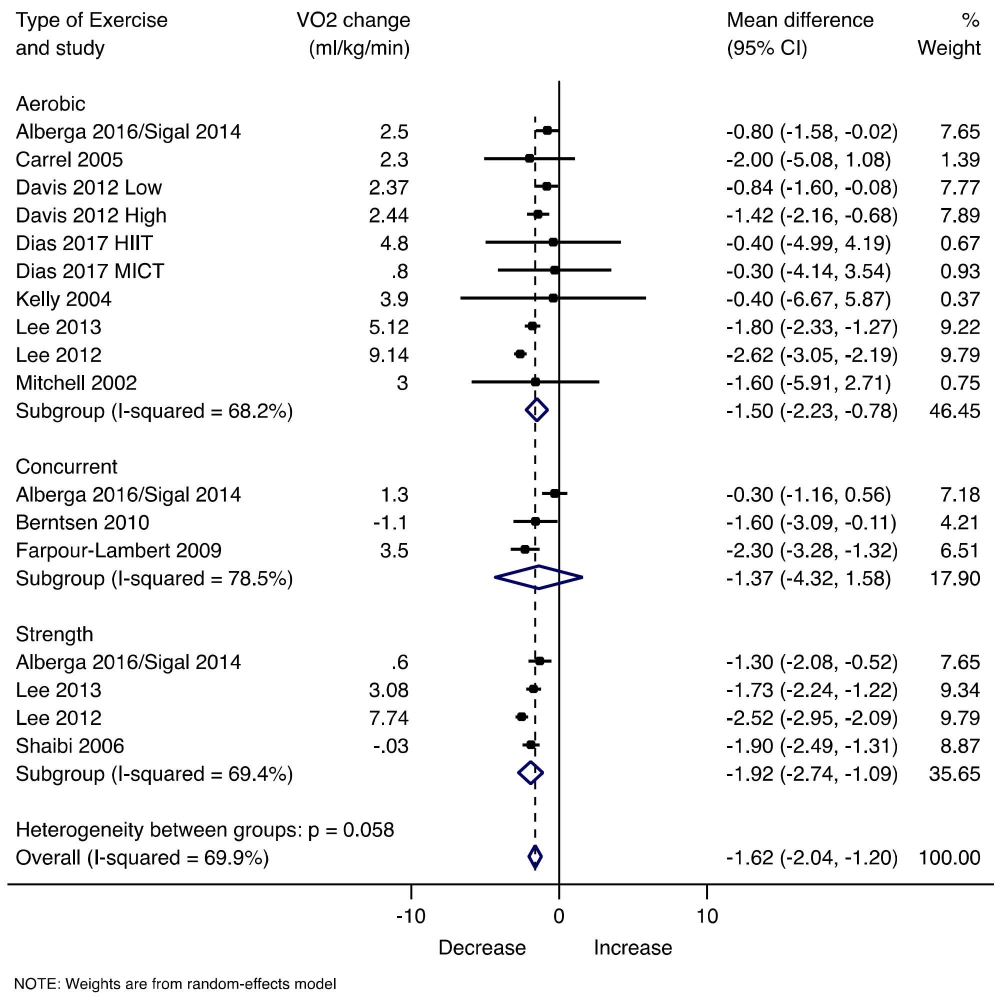
Type of Exercise and study
VO2 change (ml/kg/min)
Mean difference (95% CI)
% Weight
Aerobic
Alberga 2016/Sigal 2014 2.5 -0.80 (-1.58, -0.02) 7.65
Carrel 2005 2.3 -2.00 (-5.08, 1.08) 1.39
Davis 2012 Low 2.37 -0.84 (-1.60, -0.08) 7.77
Davis 2012 High 2.44 -1.42 (-2.16, -0.68) 7.89
Dias 2017 HIIT 4.8 -0.40 (-4.99, 4.19) 0.67
Dias 2017 MICT .8 -0.30 (-4.14, 3.54) 0.93
Kelly 2004 3.9 -0.40 (-6.67, 5.87) 0.37
Lee 2013 5.12 -1.80 (-2.33, -1.27) 9.22
Lee 2012 9.14 -2.62 (-3.05, -2.19) 9.79
Mitchell 2002 3 -1.60 (-5.91, 2.71) 0.75
Subgroup (I-squared = 68.2%) -1.50 (-2.23, -0.78) 46.45
Concurrent
Alberga 2016/Sigal 2014 1.3 -0.30 (-1.16, 0.56) 7.18
Berntsen 2010 -1.1 -1.60 (-3.09, -0.11) 4.21
Farpour-Lambert 2009 3.5 -2.30 (-3.28, -1.32) 6.51
Subgroup (I-squared = 78.5%) -1.37 (-4.32, 1.58) 17.90
Strength
Alberga 2016/Sigal 2014 .6 -1.30 (-2.08, -0.52) 7.65
Lee 2013 3.08 -1.73 (-2.24, -1.22) 9.34
Lee 2012 7.74 -2.52 (-2.95, -2.09) 9.79
Shaibi 2006 -.03 -1.90 (-2.49, -1.31) 8.87
Subgroup (I-squared = 69.4%) -1.92 (-2.74, -1.09) 35.65
Heterogeneity between groups: p = 0.058
Overall (I-squared = 69.9%) -1.62 (-2.04, -1.20) 100.00
-10
0
10
Decrease
Increase
NOTE: Weights are from random-effects model

Figure S3.

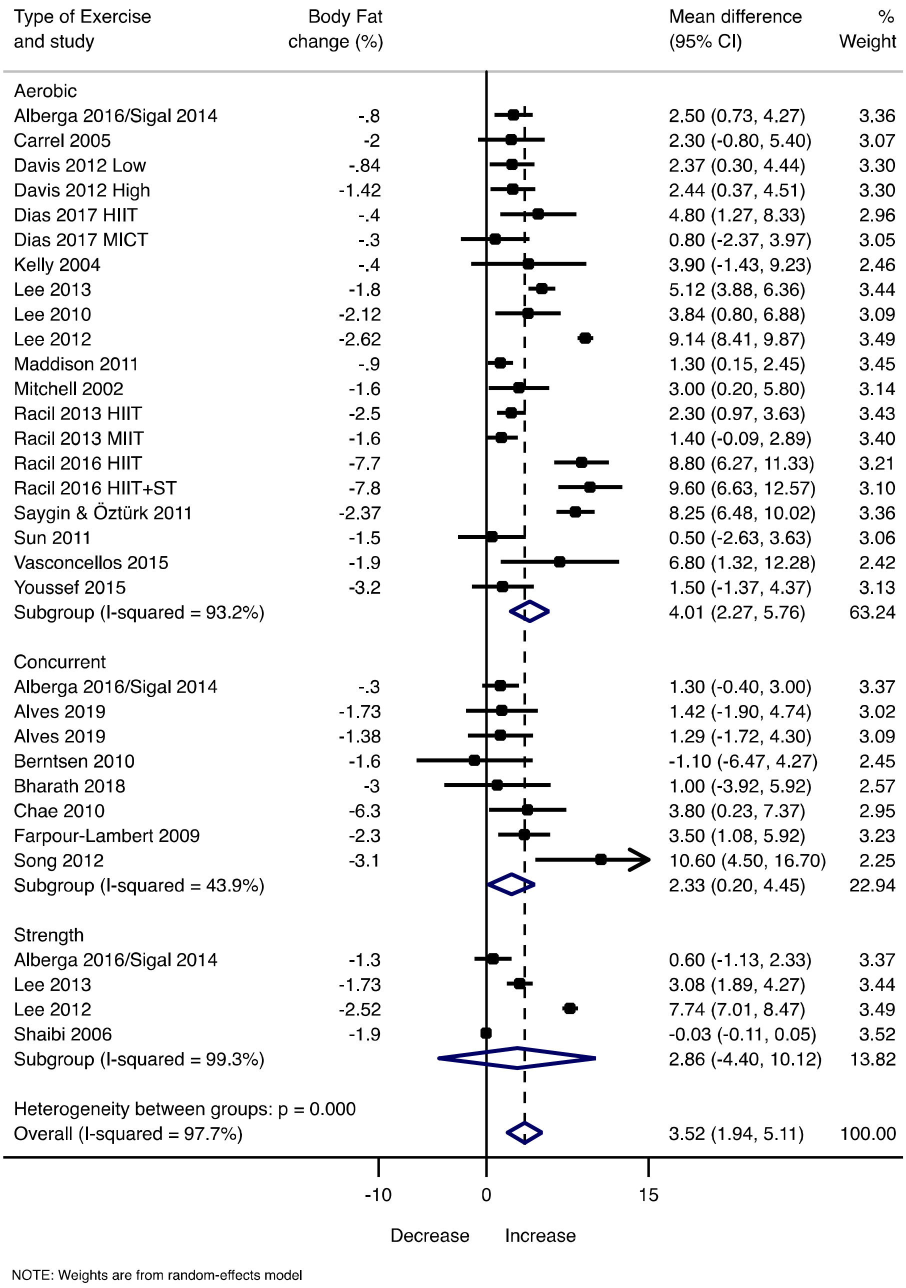
Type of Exercise and study
Body Fat change (%)
Mean difference (95% CI)
% Weight
Aerobic
Alberga 2016/Sigal 2014 -.8 2.50 (0.73, 4.27) 3.36
Carrel 2005 -2 2.30 (-0.80, 5.40) 3.07
Davis 2012 Low -.84 2.37 (0.30, 4.44) 3.30
Davis 2012 High -1.42 2.44 (0.37, 4.51) 3.30
Dias 2017 HIIT -.4 4.80 (1.27, 8.33) 2.96
Dias 2017 MICT -.3 0.80 (-2.37, 3.97) 3.05
Kelly 2004 -.4 3.90 (-1.43, 9.23) 2.46
Lee 2013 -1.8 5.12 (3.88, 6.36) 3.44
Lee 2010 -2.12 3.84 (0.80, 6.88) 3.09
Lee 2012 -2.62 9.14 (8.41, 9.87) 3.49
Maddison 2011 -.9 1.30 (0.15, 2.45) 3.45
Mitchell 2002 -1.6 3.00 (0.20, 5.80) 3.14
Racil 2013 HIIT -2.5 2.30 (0.97, 3.63) 3.43
Racil 2013 MIIT -1.6 1.40 (-0.09, 2.89) 3.40
Racil 2016 HIIT -7.7 8.80 (6.27, 11.33) 3.21
Racil 2016 HIIT+ST -7.8 9.60 (6.63, 12.57) 3.10
Saygin & Öztürk 2011 -2.37 8.25 (6.48, 10.02) 3.36
Sun 2011 -1.5 0.50 (-2.63, 3.63) 3.06
Vasconcellos 2015 -1.9 6.80 (1.32, 12.28) 2.42
Youssef 2015 -3.2 1.50 (-1.37, 4.37) 3.13
Subgroup (I-squared = 93.2%) 4.01 (2.27, 5.76) 63.24
Concurrent
Alberga 2016/Sigal 2014 -.3 1.30 (-0.40, 3.00) 3.37
Alves 2019 -1.73 1.42 (-1.90, 4.74) 3.02
Alves 2019 -1.38 1.29 (-1.72, 4.30) 3.09
Berntsen 2010 -1.6 -1.10 (-6.47, 4.27) 2.45
Bharath 2018 -3 1.00 (-3.92, 5.92) 2.57
Chae 2010 -6.3 3.80 (0.23, 7.37) 2.95
Farpour-Lambert 2009 -2.3 3.50 (1.08, 5.92) 3.23
Song 2012 -3.1 10.60 (4.50, 16.70) 2.25
Subgroup (I-squared = 43.9%) 2.33 (0.20, 4.45) 22.94
Strength
Alberga 2016/Sigal 2014 -1.3 0.60 (-1.13, 2.33) 3.37
Lee 2013 -1.73 3.08 (1.89, 4.27) 3.44
Lee 2012 -2.52 7.74 (7.01, 8.47) 3.49
Shaibi 2006 -1.9 -0.03 (-0.11, 0.05) 3.52
Subgroup (I-squared = 99.3%) 2.86 (-4.40, 10.12) 13.82
Heterogeneity between groups: p = 0.000
Overall (I-squared = 97.7%) 3.52 (1.94, 5.11) 100.00
-10
0
15
Decrease
Increase
NOTE: Weights are from random-effects model

Table S1.

	1*	2	3	4	5	6	7	8	9	10	11	Total
Alberga 2016 [14]/Sigal 2014 [15]	0	1	0	1	0	0	1	0	1	1	1	6
Alves et al. 2019 [26]	1	1	0	1	0	0	0	0	0	1	1	4
Berntsen 2010 [30]	1	1	0	1	0	0	0	1	1	1	1	6
Bharath 2018 [31]	0	1	0	1	0	0	0	1	1	1	1	6
Carrel 2005 [32]	1	1	0	1	0	0	0	1	0	1	1	5
Chae 2010 [33]	1	1	0	1	0	0	0	1	0	1	1	5
Davis 2012 [34]	1	1	1	1	0	0	0	1	1	1	1	7
Dias 2018 [35]	1	1	1	1	0	0	0	1	1	1	1	7
Farpour-Lambert 2009 [36]	1	1	1	0	0	1	1	1	1	1	1	8
Kelly 2004 [16]	1	1	0	1	0	0	1	1	0	1	1	6
Lee 2013 [17]	1	1	0	1	0	0	0	0	1	1	1	5
Lee 2010 [37]	1	1	0	1	0	0	0	0	0	1	1	4
Lee 2012 [18]	1	1	1	1	0	0	0	1	1	1	1	7
Maddison 2011 [19]	1	1	1	1	0	0	0	0	1	1	1	6
Mitchell 2002 [21]	1	1	0	0	0	0	0	0	0	1	1	3
Racil 2013 [22]	1	1	0	1	0	0	0	1	0	1	1	5
Racil 2016 [23]	0	1	0	1	0	0	0	1	0	1	1	5
Saygın 2011 [24]	1	1	0	1	0	0	0	1	0	1	1	5
Shaibi 2006 [25]	1	1	0	1	0	0	0	0	0	1	1	4
Song 2012 [27]	1	1	0	1	0	0	0	1	0	1	1	5
Sun 2011 [20]	1	1	0	1	0	0	0	1	0	1	1	5
Vasconcellos 2016 [28]	1	1	1	1	0	0	0	0	0	1	1	5
Youssef 2015 [29]	1	1	0	1	0	0	0	0	1	1	1	5

*Item #1 is not used to calculate final rating

1. eligibility criteria were specified; 2. subjects were randomly allocated to groups (in a crossover study, subjects were randomly allocated an order in which treatments were received); 3. allocation was concealed; 4. the groups were similar at baseline regarding the most important prognostic indicators; 5. there was blinding of all subjects; 6. there was blinding of all therapists who administered the therapy; 7. there was blinding of all assessors who measured at least one key outcome; 8. measures of at least one key outcome were obtained from more than 85% of the subjects initially allocated to groups; 9. all subjects for whom outcome measures were available received the treatment or control condition as allocated or, where this was not the case, data for at least one key outcome was analyzed by “intention to treat”; 10. the results of between-group statistical comparisons are reported for at least one key outcome; 11. the study provides both point measures and measures of variability for at least one key outcome.
